# Repeated Anodal Transcranial Direct Current Stimulation (RA-tDCS) over the Left Frontal Lobe Increases Bilateral Hippocampal Cell Proliferation in Young Adult but Not Middle-Aged Female Mice

**DOI:** 10.3390/ijms24108750

**Published:** 2023-05-14

**Authors:** Stéphanie Dumontoy, Bahrie Ramadan, Pierre-Yves Risold, Solène Pedron, Christophe Houdayer, Adeline Etiévant, Lidia Cabeza, Emmanuel Haffen, Yvan Peterschmitt, Vincent Van Waes

**Affiliations:** 1 Laboratoire de Recherches Intégratives en Neurosciences et Psychologie Cognitive, Université de Franche-Comté, F-25000 Besançon, France; 2 Porsolt SAS, F-53940 Le Genest-Saint-Isle, France

**Keywords:** non-invasive brain stimulation, tDCS, neurogenesis, BrdU, hippocampus, mice

## Abstract

Repeated anodal transcranial direct current stimulation (RA-tDCS) is a neuromodulatory technique consisting of stimulating the cerebral cortex with a weak electric anodal current in a non-invasive manner. RA-tDCS over the dorsolateral prefrontal cortex has antidepressant-like properties and improves memory both in humans and laboratory animals. However, the mechanisms of action of RA-tDCS remain poorly understood. Since adult hippocampal neurogenesis is thought to be involved in the pathophysiology of depression and memory functioning, the purpose of this work was to evaluate the impact of RA-tDCS on hippocampal neurogenesis levels in mice. RA-tDCS was applied for 20 min per day for five consecutive days over the left frontal cortex of young adult (2-month-old, high basal level of neurogenesis) and middle-aged (10-month-old, low basal level of neurogenesis) female mice. Mice received three intraperitoneal injections of bromodeoxyuridine (BrdU) on the final day of RA-tDCS. The brains were collected either 1 day or 3 weeks after the BrdU injections to quantify cell proliferation and cell survival, respectively. RA-tDCS increased hippocampal cell proliferation in young adult female mice, preferentially (but not exclusively) in the dorsal part of the dentate gyrus. However, the number of cells that survived after 3 weeks was the same in both the Sham and the tDCS groups. This was due to a lower survival rate in the tDCS group, which suppressed the beneficial effects of tDCS on cell proliferation. No modulation of cell proliferation or survival was observed in middle-aged animals. Our RA-tDCS protocol may, therefore, influence the behavior of naïve female mice, as we previously described, but its effect on the hippocampus is only transient in young adult animals. Future studies using animal models for depression in male and female mice should provide further insights into RA-tDCS detailed age- and sex-dependent effects on hippocampal neurogenesis.

## 1. Introduction

Neurogenesis is the complex process through which new neurons are generated and proliferated from brain stem cells, including their differentiation, before joining different final cellular networks [1,2]. In adults, this process takes place in two distinct brain regions: the subventricular zone (SVZ) of the lateral ventricles and the subgranular zone (SGZ) of the dentate gyrus (DG) of the hippocampus [3]. Several studies have shown that hippocampal neurogenesis decreases significantly with age [3,4,5]. The literature suggests the existence of a dorsoventral functional gradient inside this structure. The dorsal hippocampus may be involved mainly in spatial learning and memory, while the ventral hippocampus may be associated with limbic functions, such as emotional regulation [6,7,8,9,10]. Alterations in hippocampal adult neurogenesis have been associated with major depressive disorder (see, for example, [11,12,13]). Patients suffering from depression show a significant decrease in newborn neurons along with hippocampal atrophy [14]. The integrity of the hippocampus is considered essential for a healthy mood and cognition. Preclinical studies have also shown that suppressing neurogenesis reduces the ability of rodents to form new memories in a spatial-related paradigm [15], suggesting an impaired encoding of new information [16]. Adult hippocampal neurogenesis in both humans and rodents can be pharmacologically stimulated, for instance with antidepressant (AD) drugs [16,17]. Enhanced neurogenesis might be beneficial for the treatment of mood disorders, such as anxiety and depression, as well as for improving memory performance.

Transcranial direct current stimulation (tDCS) is a safe, non-invasive neuromodulatory technique proposed as a therapeutic tool for the treatment of several psychiatric conditions, such as depression and memory deficits [18,19,20,21,22,23,24]. Using a weak electric current flowing between two external electrodes placed on the scalp, tDCS induces long-lasting changes in cortical excitability [19,25,26]. However, the mechanisms underlying its effects remain poorly understood. In an effort to better identify the neurobiological processes involved, we have previously set up a tDCS protocol that can be used in mice [27,28,29,30,31,32,33]. We have shown that repeated sessions of anodal tDCS (RA-tDCS) have AD-like properties in naïve [29] and in depressed (i.e., in CORT-treated) [28] young adult mice and significantly improve working memory in naïve young adult mice [29].

Since adult hippocampal neurogenesis is thought to be involved in the pathophysiology of depression and memory functioning, the purpose of this work was to evaluate the impact of RA-tDCS on hippocampal neurogenesis levels in naïve female mice. Here, we hypothesize that an increase in hippocampal adult neurogenesis might contribute to the beneficial effects of RA-tDCS on mood and cognition previously observed by our team [28,29]. The impact of RA-tDCS on neurogenesis in the DG of the hippocampus has been explored only recently. For example, Pikhovych and collaborators have studied the impact of tDCS in the SVZ and showed that brain-resident neuronal stem cells are affected by both anodal and cathodal tDCS, leading to an increase in young neuroblasts in naïve C57BL/6J male mice [34]. More recently, Yu and colleagues conducted an elegant and comprehensive study exploring the effects of RA-tDCS on the hippocampus, demonstrating increased cell proliferation, differentiation, and survival in naïve C57BL/6J male mice. However, their stimulation protocol differed slightly from ours, including variations in the age, sex, and strain of the mice, as well as in electrode placement and the total number of days of stimulation [35]. These differences prevent a direct comparison of their results with the behavioral data we have previously collected using our protocol of stimulation in naïve female Swiss mice [29].

Since neurogenesis in the hippocampus decreases significantly with age, we evaluated in the present work the effects of RA-tDCS over the left frontal lobe on cell proliferation, survival, and differentiation in the DG of the dorsal and ventral hippocampus in naïve Swiss female young adult (two-month-old) and middle-aged (ten-month-old) mice to evaluate the potential of our RA-tDCS protocol to reduce the age-induced decline of neurogenesis.

## 2. Results

### 2.1. RA-tDCS Does Not Differentially Impact Cell Proliferation and Cell Survival across Hemispheres

Cell proliferation was assessed 1 day and cell survival 3 weeks post-BrdU injections. Although the stimulation electrode was positioned asymmetrically on the skull (1 mm left of the bregma), there was no difference between the left (stimulated) and the right (contralateral) hemisphere in the number of bromodeoxyuridine (BrdU^+^) cells, with or without RA-tDCS stimulation (Table 1). That is, there was neither a significant effect of the hemisphere on the number of BrdU^+^ cells (ipsilateral vs. contralateral) nor an interaction between the hemisphere and the electrical stimulation (Table 1). Data from both hemispheres were, therefore, averaged for the rest of this study.

### 2.2. RA-tDCS Increases Cell Proliferation in Young Adult Mice but Does Not Modulate the Number of Cells That Survived 3 Weeks Post-Stimulation

**Figure 1:**
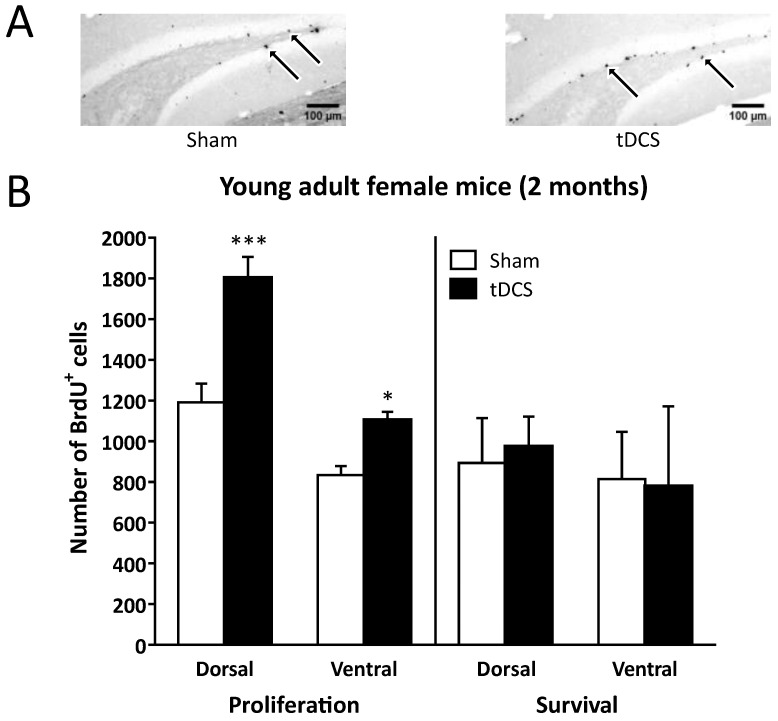
(**A**) A representative immunohistochemical image of mouse dorsal hippocampal DG sections from the Sham and tDCS groups. The black arrows indicate BrdU^+^ cells. Scale bar: 100 µm. (**B**) In young adult (two-month-old) female mice, RA-tDCS increased cell proliferation in the DG of the dorsal and the ventral hippocampus (quantified one day after the first BrdU injection). The number of cells that survived 3 weeks post-stimulation was not affected by RA-tDCS. * *p* < 0.05, *** *p* < 0.001 vs. Sham.

RA-tDCS significantly increased the number of BrdU^+^ cells in naïve young adult mice (proliferation) but did not affect the number of cells that survived 3 weeks post-stimulation (Figure 1). Concerning cell proliferation, repeated measures analysis of variance (RM-ANOVA) revealed that there were significant effects from the electrical stimulation (tDCS vs. Sham; F_(1, 18)_ = 31.92, *p* < 0.001) from the region (dorsal hippocampus [dHi] vs. ventral hippocampus [vHi]; F_(1, 18)_ = 53.96, *p* < 0.001) and the stimulation × region interaction (F_(1, 18)_ = 5.65, *p* < 0.05). Although both the dorsal and ventral regions of the DG were significantly impacted by RA-tDCS, Newman–Keuls (NK) post hoc analyses revealed that the increase in cell proliferation was more robust in the dHi (51.38 ± 10.68% increase, *p* < 0.001) than in the vHi (32.42 ± 8.72% increase, *p* < 0.05).

Concerning cell survival, RM-ANOVA did not reveal any significant effect (stimulation effect: F_(1, 15)_ = 0.07, *p* = 0.79; region effect: F_(1, 15)_ = 2.27, *p* = 0.15; stimulation × region interaction: F_(1, 15)_ = 0.41, *p* = 0.53). The ratio of cell survival in the dHi was 75.0 ± 6.2% for the Sham group, while it dropped to 54.1 ± 2.9% for the tDCS group. In the vHi, the ratio of cell survival was 97.6 ± 9.3% for the Sham group and 70.5 ± 12.5% for the tDCS group.

### 2.3. RA-tDCS Has No Significant Effect on Cell Proliferation and Cell Survival in Middle-Aged Mice

**Figure 2:**
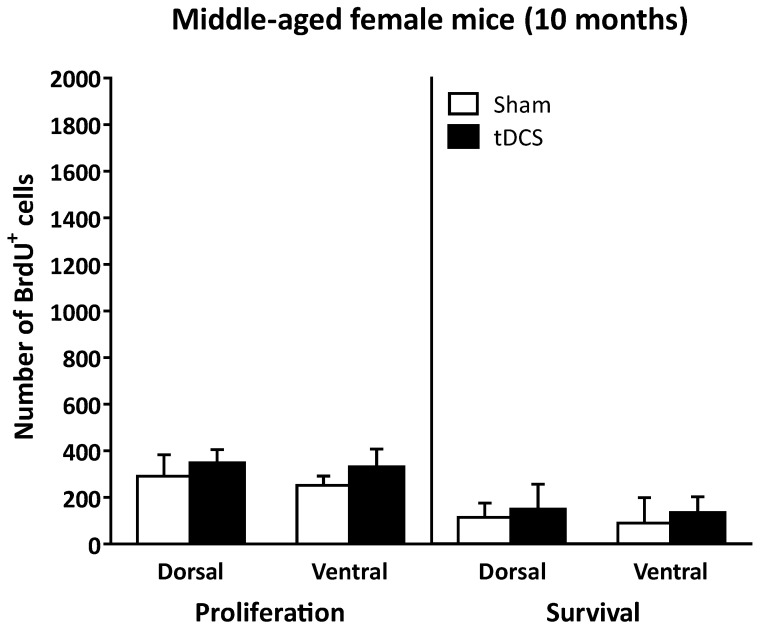
In naïve middle-aged (10-month-old) female mice, neither cell proliferation in the DG of the hippocampus (quantified one day after the first BrdU injection) nor cell survival (quantified 3 weeks after the first BrdU injection) was affected by RA-tDCS.

RA-tDCS did not significantly impact cell proliferation and cell survival in the DG of the hippocampus of middle-aged (10-month-old) female mice (Figure 2). Concerning cell proliferation, RM-ANOVA did not reveal any significant effect (stimulation effect: F_(1, 16)_ = 0.54, *p* = 0.47; region effect: F_(1, 16)_ = 0.41, *p* = 0.53; stimulation × region interaction: F_(1, 16)_ = 0.06, *p* = 0.80).

Similarly, cell survival was not significantly impacted in middle-aged female mice (stimulation effect: F_(1, 22)_ = 1.58, *p* = 0.22; region effect: F_(1, 22)_ = 1.85, *p* = 0.19; stimulation × region interaction: F_(1, 22)_ = 0.10, *p* = 0.76). The ratio of cell survival was similar in the dHi (39.1 ± 6.1% and 42.5 ± 9.1% for the Sham and tDCS groups, respectively) and in the vHi (35.4 ± 12.5% and 40.1 ± 6.2% for the Sham and tDCS groups, respectively).

### 2.4. RA-tDCS Stimulation Does Not Affect Cell Differentiation in Either Young Adult or Middle-Aged Female Mice

**Figure 3:**
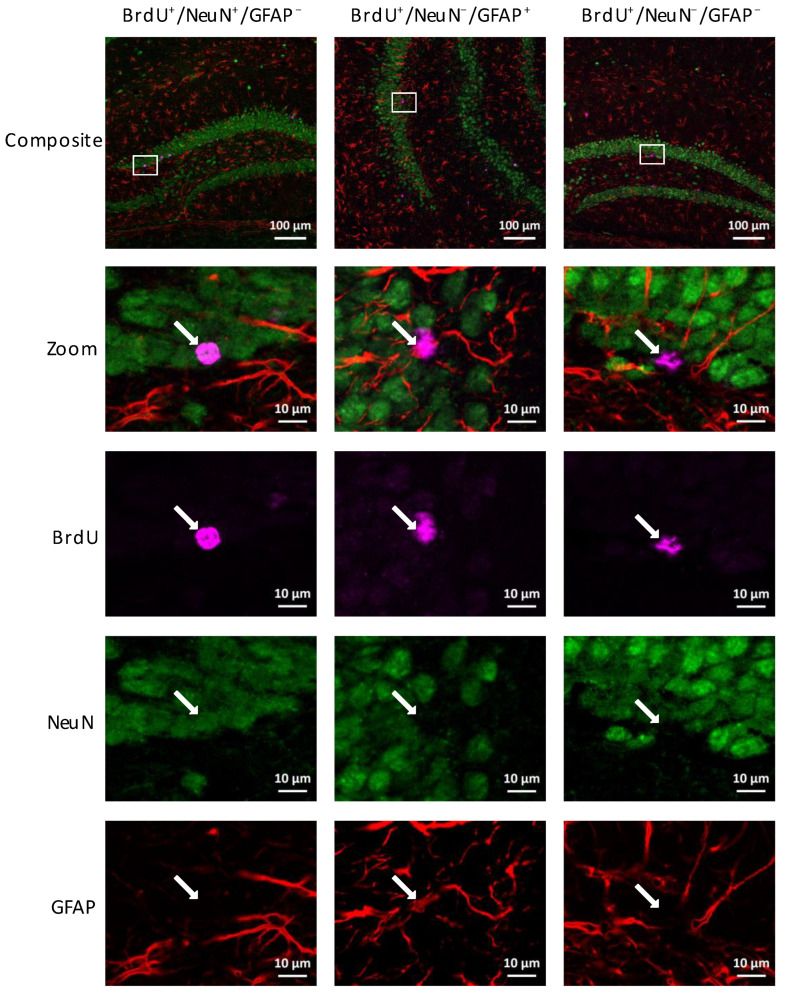
Representative immunofluorescence images of dorsal and ventral hippocampal DG sections from young adult Sham-treated mice triple stained for BrdU (purple), NeuN (green), and GFAP (red) 3 weeks after BrdU injections. The white arrows indicate co-labeled cells. Scale bars: 100 µm for the composite images and 10 µm for the others.

RA-tDCS had no effect on the differentiation of newborn cells that survived. Regardless of the region studied and the age of the mice, the proportion of neurons, astrocytes, and undifferentiated cells were similar in the tDCS and Sham groups (for more details, see Figure 3 and Supplementary Data Table S1).

**Figure 4:**
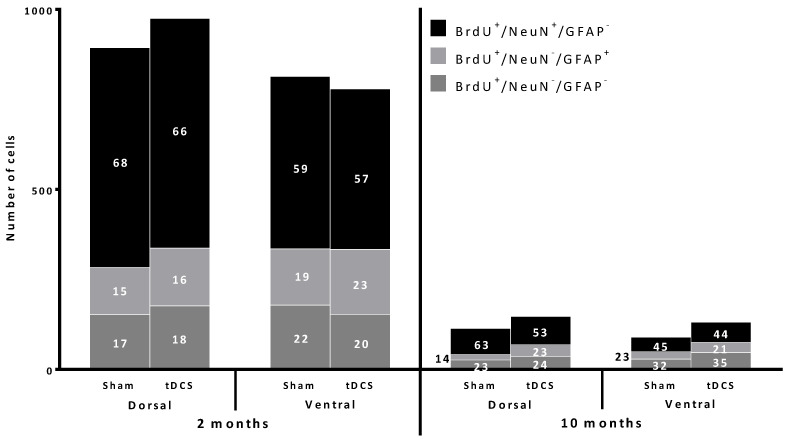
RA-tDCS does not influence the differentiation of newly formed BrdU^+^ cells (absolute number of cells and percentages of cell differentiation in terms of the estimated number of cells). Newborn cells survived display co-immunolabeling when they differentiated into neurons (BrdU^+^/NeuN^+^/GFAP^−^) or astrocytes (BrdU^+^/ NeuN^−^/GFAP^+^). BrdU^+^/NeuN^−^/GFAP^−^ cells are considered undifferentiated.

In the dHi of Sham young adult female mice, 68.4 ± 7.1% of the newly formed cells differentiated into neurons and 14.5 ± 4.4% into astrocytes, and the remaining 17.1 ± 3.5% were undifferentiated. In the tDCS group, the percentages were 65.4 ± 6.6%, 16.4 ± 3.2%, and 18.2 ± 1.7%, respectively (Figure 4).

In the vHi of Sham young adult female mice, 58.9 ± 5.7% of the newly formed cells differentiated into neurons and 19.2 ± 3.8% into astrocytes, and the remaining 21.9 ± 2.7% were undifferentiated. In the tDCS group, the percentages were 57.2 ± 11.4%, 23.3 ± 3.7%, and 19.5 ± 4.2%, respectively (Figure 4).

In the dHi of Sham middle-aged female mice, 63.6 ± 12.7% of the newly formed cells differentiated into neurons and 13.6 ± 4.4% into astrocytes, and the remaining 22.7 ± 4.6% were undifferentiated. In the tDCS group, the percentages were 53.5 ± 17.3%, 22.8 ± 5.0%, and 23.7 ± 4.3%, respectively (Figure 4).

In the vHi of Sham middle-aged female mice, 45.4 ± 14.7% of the newly formed cells differentiated into neurons and 22.7 ± 6.6% into astrocytes, and the remaining 31.9 ± 7.8% were undifferentiated. In the tDCS group, the percentages were 43.4 ± 7.9%, 21.1 ± 5.4%, and 35.4 ± 7.4%, respectively (Figure 4). 

## 3. Discussion

In this study, we demonstrate for the first time that RA-tDCS differently influences cell proliferation in the DG of the hippocampus depending on the age of the mice. Electrical stimulation boosts cell proliferation of young adult (2-month-old) female mice but has no impact in middle-aged (10-month-old) female mice. Moreover, we found that our RA-tDCS stimulation protocol affects neither the number of cells that survived 3 weeks post-stimulation nor cell differentiation in female mice, whatever their age. Using exactly the same RA-tDCS protocol (i.e., anodal stimulation, frontal lobe, 0.2 mA, 5 consecutive days, twice a day for 20 min, a fade-in and fade-out of 10 s, at least 6 h between stimulations, and the same electrode placement), we have previously shown that repeated anodal stimulations of the left frontal cortex induce antidepressant and pro-memory effects in 2- to 5-month-old adult naïve female Swiss mice [19,29]. Our findings suggest that the behavioral effects of RA-tDCS previously described by our team [19,29] may not involve a modulating effect of RA-tDCS on hippocampal neurogenesis. Differently, Pikhovych’s team studied the effect of tDCS for 10 days (2 × 5 days; one stimulation per day; right frontal cortex) in naïve 10- to 12-week-old C57BL/6J male mice with an anodal or cathodal constant current (0.25 mA) on the number of doublecortin-positive cells (DCX^+^, neuroblasts) in the SVZ. They showed a bilateral increase in neuroblast numbers after cathodal stimulation and an ipsilateral (right hemisphere) increase after anodal stimulation. However, they did not observe an effect on cell proliferation in the SVZ (number of BrdU^+^ cells) [34]. Concerning the DG of the hippocampus, Yu et al. [35] have recently explored the impact of RA-tDCS (0.25 mA) for 10 days (2 × 5 days; one stimulation per day; midline caudal cortex) in naïve 8- to 12-week-old C57BL/6J male mice. They reported an increase in proliferation (number of BrdU^+^ cells), differentiation for all types of neural stem or progenitor cells (number of BrdU^+^/GFAP^+^/SRY-box transcription factor 2 positive [Sox2^+^], BrdU^+^/GFAP^−^/Sox2^+^, BrdU^+^/Sox2^+^/DCX^+^, and BrdU^+^/Sox2^−^/DCX^+^ cells), and survival (number of immature [DCX^+^] and mature neurons [NeuN^+^]) [35]. These effects on neurogenesis were associated with behavioral outcomes (i.e., enhanced performance on a contextual fear discrimination task). Interestingly, this enhancement was prevented by blocking neurogenesis using the DNA-alkylating agent temozolomide [35]. There are many differences between our study and that of Yu and colleagues that may explain our different results on cell survival. To reconcile these discrepancies, it would be interesting to determine the importance of the mouse strain used (C57BL/6J vs. Swiss in our study), the gender (male vs. female), the brain area stimulated (the hippocampus vs. the left frontal lobe), the protocol of stimulation (20 min at 0.25 mA once daily for 10 days in total vs. 20 min at 0.2 mA twice daily for 5 consecutive days), the consciousness state during the stimulation (slightly anesthetized vs. awake), and the age (2- to 3-month-old male mice vs. 2- and 10-month-old female mice in our study) in the potential effects of tDCS on neurogenesis.

### 3.1. The Absence of Asymmetric Effects of tDCS between the Left and the Right Hippocampus

The bilateral effects of RA-tDCS following unilateral stimulation confirm our previous data. For example, we have shown that the same tDCS protocol (unilateral, repeated anodal stimulation over the left frontal lobe) bilaterally induces Zif268 expression in the basal ganglia (3 weeks after the last electrical stimulation) [30]. Furthermore, our tDCS protocol was also able to bilaterally reduce the cocaine-induced activation of corticostriatal circuits (Zif268) [30]. This indicates that our protocol of stimulation produces effects on both hemispheres. We do not yet know the mechanisms underlying this bilateral effect. Recently, we obtained an interesting result by mapping (quantification of cFos expression by immunohistochemistry) the brain areas affected by a single 20 min stimulation vs. multiple (10) 20 min stimulations, as used in our present experiment. Following a single stimulation, we observed a unilateral effect on the left side under the electrode, while repeated stimulations caused a bilateral effect in the two hemispheres (data not published). We aim to conduct complementary experiments to determine the impact of repeated stimulation on the brain areas activated by an electrical current after 1, 2, 5, 7, or 10 stimulation sessions to determine the differential impact of single vs. repeated stimulation sessions on brain activation. These results will allow us to better describe the kinetics of the transition from a unilateral to a bilateral effect following repeated electrical stimulation.

### 3.2. RA-tDCS Affects Young Adult and Middle-Aged Mice Differently

We based our study on two significant time points characterized by high (2-month-old, young adult) and low (10-month-old, middle-aged) rates of hippocampal neurogenesis according to Klempin and colleagues’ work [10]. This team used a similar protocol to assess neurogenesis and showed a drastic reduction in proliferation and survival rates in the hippocampus of 12-month-old female C57BL/6N mice compared with 80-day-old mice. For the sake of direct comparison, we used the same animals (female Swiss young adult mice) as in previously published studies (regarding the effects of RA-tDCS on memory and symptoms associated with depression [29]). In the present study, we found a 76% and 70% reduction in cell proliferation rates and an 87% and 89% reduction in cell survival rates in the Sham group in the ventral and the dorsal hippocampus, respectively, between 2-month-old (young adult) and 10-month-old (middle-aged) mice. Although 10-month-old mice are not considered aged animals, the decline in neurogenesis with age has already started at this point in their life cycle.

It is interesting to note that the increase in cell proliferation induced by tDCS in young adult mice (2-month-old, +51% in the dHi, +32% in the vHi) is not found in middle-aged mice (10-month-old), for which cell proliferation and survival rates are already very low in the Sham group. This shows that in our experimental conditions, tDCS is less effective in modulating cell proliferation when cell proliferation and survival rates are low, which is unfortunate because, from a clinical perspective, this is the time point at which we would have wanted to see the most marked effects. This age-related difference can be explained by the observation of a progressive (≈ 80%) decline in the hippocampal neurogenesis level (proliferation and survival rates) that has also been reported in Fischer 344 female rats [36] and C57BL/6 male mice [10,37]. Stimulating conditions, such as learning, exposure to an enriched environment, and physical activity, can partially reverse the age-related decline in neurogenesis [10,38]. For example, Klempin and colleagues [10] reported that wheel-running exercise increases cell proliferation in the hippocampus (BrdU^+^ cell numbers) and neurogenesis (BrdU^+^/DCX^+^ cells). This result was observed, to some extent, in mice of all ages, but the improvement was smaller in older mice. In fact, physical activity increased proliferation by 138% and 73% in the hippocampus of 1.5-month-old and 12-month-old female C57BL/6N mice [10]. RA-tDCS increases proliferation by 44% in the whole hippocampus of naïve young adult (2-month-old) female mice. Thus, it seems that our RA-tDCS protocol improves the levels of cell proliferation in the hippocampus, but these effects are less robust than the effects produced by physical exercise, which would explain why the effects are no longer visible in older animals. Age could be a limiting factor for the therapeutic effects of tDCS, at least with respect to cell proliferation. It would be interesting to evaluate the impact of tDCS on memory performance and behaviors associated with depression, as well as on cell proliferation in the hippocampus, at different ages. Correlations between cell proliferation in the hippocampus and behavioral effects assessed in the same animals would provide a better understanding of the mechanisms of action of RA-tDCS.

### 3.3. The Number of New Cells in the Hippocampus of Young Adults Is Not Affected 3 Weeks after Electrical Stimulation

Although it appears that the number of BrdU^+^ cells is identical in the DG of the Sham and tDCS animals at 3 weeks post-BrdU injection, tDCS animals present a significantly higher number of BrdU^+^ cells one day after BrdU injection. This leads to an overall higher survival rate of BrdU^+^ cells in the Sham group compared with the tDCS group, suggesting that an increased number of newborn neurons do not survive in the tDCS group, although we cannot draw a definitive conclusion. In middle-aged animals, the survival rate was not affected, supporting the hypothesis that tDCS was not toxic for hippocampal cells per se. This rather suggests that newly formed cells do not survive and that the effect of our RA-tDCS protocol on the hippocampus is only transient, at least in naïve female Swiss mice.

Despite the increased proliferation rates in young adults, our RA-tDCS protocol did not increase neurogenesis. Only naïve mice were used in this work (mice without depression-like behavior or cognitive impairment); therefore, we could not definitively conclude that our RA-tDCS protocol has no effect on neurogenesis levels. We can only deduce that it does not counteract an age-related decline in neurogenesis in naïve animals, but perhaps an improvement could take place if we used an animal model of depression (e.g., induced by a chronic oral administration of CORT).

This will have to be performed in future studies. Indeed, David’s team reported, for example, that fluoxetine (an SSRI antidepressant, AD) had no effects on hippocampal neurogenesis in naïve male mice (4- to 6-month-old), whereas it significantly increased cell proliferation (+167%) and survival (+28%) in CORT-treated animals [39]. Moreover, a recent study by Yu’s team indicates that electrical stimulation (10 days) increases neurogenesis in naïve C57BL/6J male mice. The impact of gender on the neurobiological and behavioral effects induced by RA-tDCS should be explored by using our stimulation protocol on male mice as well. The use of female rather than male mice in our study may also increase the variance because of variability in the estrous cycle. 

tDCS shows potential for use in depressed patients. However, tDCS is also utilized as a cognitive enhancer in patients without depressive disorders. In fact, our team has previously reported an improvement in spatial memory in naïve 5-month-old Swiss mice [29]. Our findings suggest, therefore, that the behavioral effects observed in naïve mice [29] may be independent of the effect of tDCS on hippocampal neurogenesis.

Exploring the effects of RA-tDCS applied at different time points should also be pursued. In fact, applying RA-tDCS two weeks after the first BrdU injections could help understand whether new cell survival rates could specifically be modulated. It would also be worth investigating whether prolonging the treatment duration (several weeks) or replicating the sessions could increase the chances of modulating neurogenesis levels using our RA-tDCS protocol. Finally, it is important to emphasize that tDCS, rather than replacing AD treatments, could be used in adjunction to increase the success rate/efficacy. Future studies on neurogenesis levels in the hippocampus will be conducted in mice receiving concurrent RA-tDCS and AD treatments or RA-tDCS and physical exercise.

## 4. Materials and Methods

### 4.1. Animals

Seventy-nine naïve Swiss female mice (Janvier Labs, Saint-Berthevin, France) were used in this study. They were group-housed (five animals per home cage) under standard laboratory conditions (12 h light/dark cycle; light on at 7 am, humidity 55 ± 10%; temperature 21 ± 0.5 °C), except during surgery recovery and electrical stimulation periods, when they were housed individually. Food and water were available ad libitum. All experiments were conducted during the light phase of the cycle.

**Figure 5:**
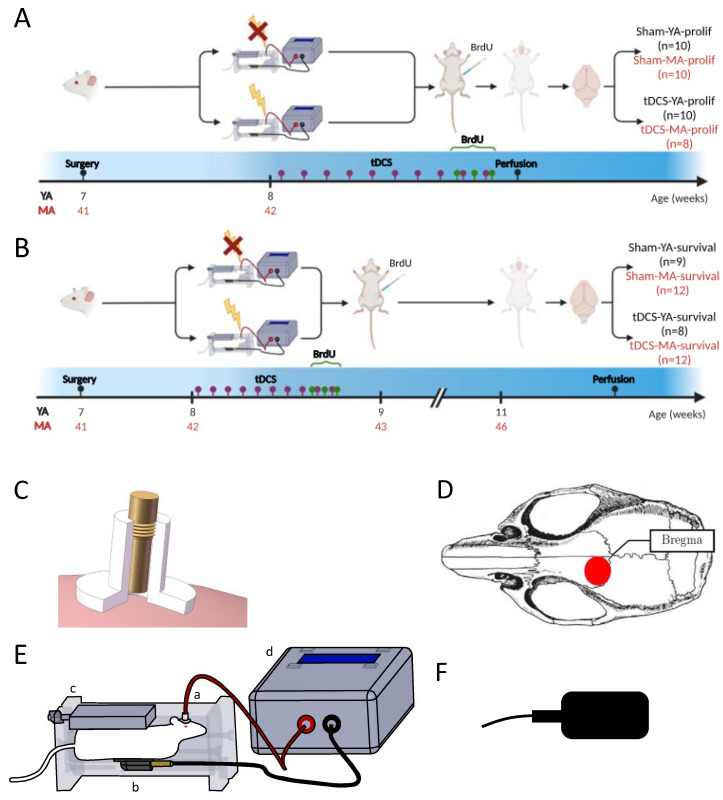
Experimental design and illustration of the tDCS device used to deliver the current. (**A**) Experimental design for measuring the effect of RA-tDCS on cell proliferation. Young adult (YA, black) and middle-aged (MA, red) female Swiss mice were stimulated with RA-tDCS for five consecutive days (2 × 20 min/day with a constant current of 0.2 mA; purple dots). Bromodeoxyuridine (BrdU, 50 mg/kg of body weight, intraperitoneal [i.p.]; green dots) was injected three times 6 h apart on the fifth day of the stimulation period. The mice received a lethal dose of pentobarbital one day after the first BrdU injection. (**B**) Experimental design for measuring the effect of RA-tDCS on cell survival and differentiation. Young adult (YA, black) and middle-aged (MA, red) female Swiss mice were stimulated with RA-tDCS for five consecutive days (2 × 20 min/day with a constant current of 0.2 mA). Bromodeoxyuridine (BrdU, 50 mg/kg of body weight, i.p.) was injected three times 6 h apart on the fifth day of the stimulation period. The mice received a lethal dose of pentobarbital 3 weeks after the first BrdU injection. (**C**) The electrode holder (internal diameter 2.1 mm) was surgically affixed to the skull and filled with a saline solution before stimulation. The stimulation electrode was screwed into the tubular plastic jacket so that it dipped into the saline solution. Only the saline solution was in contact with the skull. (**D**) The center of the electrode holder was positioned 1 mm rostral and 1 mm left of the bregma (image adapted from Paxinos and Franklin, 2007 [40]). (**E**) The mouse was placed into a custom-made restraining box. The anode (contact area 3.5 mm^2^; a and **C**) was positioned over the left frontal cortex, and the cathode (rubber-plate electrode, 9.5 cm^2^; b and **F**) was placed in contact with the ventral thorax using a custom-made restraining box (c). A constant current of 0.2 mA was applied transcranially in two sessions of 20 min per day for five consecutive days, with a linear fade-in and fade-out of 10 s, using a direct current stimulator (DC-Stimulator Plus) or an Open-tES stimulator (d) [31]. Adapted from Pedron et al. (2022) [32].

Prior to surgery, the mice were allowed one week of acclimation to the animal facility, during which time they were repeatedly handled in order to reduce manipulation-related stress (two sessions of 10 min per animal during the week). For each experiment, the animals were divided into two experimental groups (Sham or tDCS; see Figure 5A,B).

In experiment 1, cell proliferation was studied in young adult (YA: 2-month-old; Sham-YA-prolif, n = 10; tDCS-YA-prolif, n = 10) and middle-aged mice (MA: 10-month-old; Sham-MA-prolif, n = 10; tDCS-MA-prolif, n = 8) (Figure 5A).

In experiment 2, cell survival and differentiation were studied in young adult (Sham-YA-survival, n = 9; tDCS-YA-survival, n = 8) and middle-aged mice (Sham-MA-survival, n = 12; tDCS-MA-survival, n = 12) (Figure 5B).

All experimental procedures were performed in strict accordance with the Guide for the Care and Use of Laboratory Animals (NIH), the Animal Research: Reporting of In Vivo Experiments (ARRIVE) guidelines, and the European Union regulations on animal research (Directive 2010/63/EU). They were approved by the University of Franche-Comte Animal Care and Use Committee (CEBEA-58).

### 4.2. Surgery

One week before starting the stimulation protocol, a tubular plastic electrode holder (internal diameter 2.1 mm; DIXI Medical, Besançon, France; Figure 5C) was surgically affixed to each mouse’s skull, as previously described [27,28,29,30,31,32]. Briefly, the animals were anesthetized with ketamine hydrochloride/xylazine (80/12 mg/kg, intraperitoneal [i.p.] injection) and placed into a stereotaxic apparatus. The center of the electrode holder was positioned 1 mm rostral and 1 mm left of the bregma, over the left frontal cortex, and fixed with glass ionomer cement (GC^®^ Fuji I, Leuven, Belgium; Figure 5D). The animals were allowed one week to recover from the surgery.

### 4.3. Stimulation Protocol

The stimulation electrode (anode, diameter 2.1 mm; DIXI Medical, Besançon, France) was screwed into the electrode holder filled with a saline solution (0.9% NaCl). A custom-made restraining box was used to hold a larger rectangular rubber-plate electrode (cathode, 9.5 cm²; Physiomed Elektromedizin AG, Schnaittach, Germany; Figure 5E,F) against the ventral thorax, as previously described [29]. The animals were stimulated for five consecutive days (2 × 20 min/day; 5 h interstimulation interval [29,30,32]; linear fade-in and fade-out; 10 s ramp [27]), with an anodal constant current (0.2 mA) using a DC-Stimulator Plus (NeuroConn, Illmenau, Germany) or an Open-tES stimulator specifically designed for rodent research [31]. The control (Sham) animals were subjected to the same procedure (surgery, restraining box, and electrode fixation) but no current was delivered.

### 4.4. BrdU Administration

Bromodeoxyuridine (BrdU, Sigma-Aldrich, Saint-Quentin-Fallavier, France; 50 mg/kg, dissolved in 0.9% NaCl) was injected i.p. three times 6 h apart on the last day of the stimulation period (injections at 7 am, 1 pm, and 7 pm). Given the semi-conservative mode of DNA replication, applying three injections increases the likelihood that a dividing cell will incorporate a BrdU molecule [10,41].

### 4.5. Tissue Preparation

The mice received a lethal dose of pentobarbital (182.2 mg/kg; Dolethal, Vetoquinol, Lure, France) either one day (proliferation studies) or three weeks (survival studies) after the first BrdU injection. The animals were transcardially perfused first with 0.9% NaCl and then with 4% paraformaldehyde (PFA; Roth, Karlsruhe, Germany) in a 0.1 M phosphate buffer. The brains were removed, postfixed in 4% PFA overnight at 4 °C, and immersed in a 30% sucrose solution (Sigma-Aldrich, Saint-Quentin-Fallavier, France) for 24 h. The brains were then frozen using a SnapFrost^TM^ System (Excilone, Élancourt, France) and stored at −20 °C. Coronal sections of 30 μm were obtained using a microtome (Thermofisher Scientific, Microm, Brignais, France), cryoprotected (50% phosphate buffer saline [PBS], 25% ethylene glycol, and 25% glycerol; Roth, Karlsruhe, Germany), and stored at −20 °C until processed. Brain sections from −0.94 mm to −3.88 mm were selected (relative to the bregma [40]). Sections located between −0.94 mm and −2.80 mm (excluded) were considered the dorsal hippocampus (dHi), and those between −2.80 mm and −3.88 mm, the ventral hippocampus (vHi) [40]. Note that the ventral portion of the DG between −2.80 mm and −3.88 mm is located beneath the rhinal fissure, while the portion of the DG over the fissure is considered the middle DG. The middle DG was included in the ventral counts.

### 4.6. Immunohistochemistry

Before staining, the selected free-floating sections were treated with a hydrogen peroxide solution (0.1 M PBS + 0.6% H_2_O_2_ 30%; Sigma-Aldrich, Saint-Quentin-Fallavier, France) in order to block endogenous peroxidase activity that could lead to non-specific background staining. DNA denaturalization was carried out by immersing the tissues in 2-N-hydrochloric acid (HCl) for 30 min at 50 °C and then rinsing in a 0.1 M borate buffer at pH 8.5. Next, the sections were exposed to donkey serum in 0.3% PBS-Triton (PBS + 0.3% Triton X100; PBS-T) and then incubated overnight at 4 °C with an anti-BrdU primary antibody (rat, 1:500; Abcam, Paris, France) diluted in PBS-T. The tissues were then incubated for 2 h at room temperature with a biotinylated anti-rat IgG secondary antibody solution (donkey, 1:500; Sigma-Aldrich, Saint-Quentin-Fallavier, France). Signal amplification was conducted using an avidin horseradish peroxidase complex (ABC Elite kit, Vector Laboratories, Newark, NJ, United States), and 3,3′-diaminobenzidine tetrahydrochloride (DAB; Sigma-Aldrich, Saint-Quentin-Fallavier, France) was used as the chromogen. The brain sections were then dehydrated with alcohol, cleared with xylene, and coverslipped with Canada balsam (Roth, Karlsruhe, Germany).

### 4.7. Immunofluorescence

Before staining, the tissues’ DNA was denatured, as described above. To study the fate of newborn cells, the sections were incubated overnight at 4 °C with several primary antibody solutions: anti-BrdU (bromodeoxyuridine, rat, 1:500; Abcam, Paris, France), anti-NeuN (neuronal nuclear antigen, mouse, 1:1000; Millipore, Molsheim, France), and anti-GFAP (glial fibrillary acidic protein, rabbit, 1:1000; Millipore, Molsheim, France). The following secondary antibody solutions were then used at room temperature for 4 h: anti-rat IgG Cy3-conjugated (goat, 1:1000; Invitrogen, Waltham, MA, United States), anti-mouse Alexa647-conjugated (goat, 1:1000; Invitrogen, Waltham, MA, United States), and anti-rabbit 488-conjugated (goat, 1:1000; Invitrogen, Waltham, MA, United States). The brain sections were then coverslipped with a glycerin + PBS-T solution (60/40%). 

### 4.8. Image Acquisition and Analyses

We analyzed an average of eight slices per animal, with four corresponding to the dorsal hippocampus (dHi) and four corresponding to the ventral hippocampus (vHi). The sections were collected at approximately the same coordinates along the entire length of the hippocampus. These coordinates were visually verified under a microscope and compared with the atlas of Paxinos and Franklin [40]. Images from the DAB-stained brain sections were acquired with the 20× objective of an optical microscope (Olympus BX51, Rungis, France) equipped with a digital camera (Olympus DP50, Rungis, France) using the AnalySIS 3.1 software (version 3.1, Soft Imaging System, Olympus, Rungis, France). The number of BrdU^+^ cells was counted bilaterally (left and right hippocampus) in the DG to assess the proliferation rate. Regarding the immunofluorescence method, images were acquired with the 20× objective of the epifluorescence microscope ApoTome.2 (Axio Imager Zeiss Z2, Zeiss, Rueil Malmaison, France) equipped with a digital camera (Hamamatsu C11440, Zeiss, Rueil Malmaison, France) using the Axio Imager.Z2 software (version Zen 2.0.0.0, Zeiss, Rueil Malmaison, France). The number of BrdU^+^ cells was counted bilaterally in the DG to assess the survival rate. In order to determine the phenotype of the surviving BrdU^+^ cells, co-labeled cells were counted as follows: BrdU^+^/NeuN^+^/GFAP^−^ cells were counted as new neurons; BrdU^+^/NeuN^−^/GFAP^+^ cells as new astrocytes; and BrdU^+^/NeuN^−^/GFAP^−^ cells as undetermined new cells.

The average cell number per slice (BrdU^+^, NeuN^+^, or GFAP^+^) was measured over the entire DG and reported relative to the total size of the hippocampus studied. To obtain the number of cells in the hippocampus, the following method was used:

$$\frac{Size\;of\;hippocampus\times Average\;cell\;number\;per\;slice}{Slice\;thickness\;\left(0.03\;mm\right)\;}$$

For the size of the hippocampus, we used 1.86 mm for dHi and 1.08 mm for vHi.

### 4.9. Statistical Analysis

Data are presented as mean ± standard error of the mean (SEM). Significance was set at *p* < 0.05. The absence of asymmetric effects of tDCS between the left and the right hippocampus was investigated by repeated measures analysis of variance (RM-ANOVA), with stimulation (Sham, tDCS) as the within-subject factor, region (dHi, vHi), hemisphere (ipsilateral [Ipsi, left], and contralateral [Contra, right]) as the between-subject factors (Table 1), and the number of BrdU^+^ cells as the dependent variable. Further analysis with pooled hemispheres to study the effects of tDCS was also performed using RM-ANOVA, this time with stimulation (Sham, tDCS) as the within-subject factor, region (dHi, vHi) as the between-subject factor, and the number of BrdU^+^ cells as the dependent variable. Newman–Keuls (NK) post hoc tests were used to describe differences between individual groups. Comparisons of the percentages for cell differentiation were assessed using a chi-square (Χ^2^) test (Table S1).

## 5. Conclusions

The present study is a brief report that yielded promising results. We show that RA-tDCS modulates cell proliferation in the hippocampus of naïve young adult female Swiss mice, suggesting that unilateral electrical stimulation has bilateral physiological effects on the hippocampus. We also found, for the first time, that RA-tDCS has an age-dependent effect on cell proliferation in the hippocampus. Further experiments are needed to determine whether our RA-tDCS protocol modulates hippocampal neurogenesis in an animal model of depression and to better understand the neurobiological mechanisms involved. These additional data will help accelerate the development of this technique for use in humans to treat depression and cognitive disorders.

## Figures and Tables

**Table 1:** The absence of asymmetric effects of tDCS between the left and the right hippocampus was investigated by repeated measures analysis of variance (RM-ANOVA). Stim: electrical stimulation; Region: dorsal vs. ventral hippocampus; Hem: hemisphere, ipsilateral vs. contralateral. Significant effects are indicated in bold.

		2 months				RM-ANOVA	10 months				RM-ANOVA
		Dorsal		Ventral			Dorsal		Ventral		
		Ipsi	Contra	Ipsi	Contra		Ipsi	Contra	Ipsi	Contra	
Proliferation	Sham	599.3 ± 46.9	591.8 ± 59.8	406.4 ± 44.3	432.0 ± 14.3	**Stim *p* < 0.001** **Region *p* < 0.001** Hem *p* = 0.59 **Region × Stim *p* < 0.05** Hem × Stim *p* = 0.85 Region × Hem *p* = 0.69 Region × Hem × Stim *p* = 0.91	148.8 ± 44.7	141.2 ± 48.2	133.3 ± 30.6	112.8 ± 24.8	Stim *p* = 0.48 Region *p* = 0.44 Hem *p* = 0.94 Region × Stim *p* = 0.78 Hem × Stim *p* = 0.38 Region × Hem *p* = 0.33 Region × Hem × Stim *p* = 0.16
	tDCS	896.0 ± 67.9	905.5 ± 53.1	549.5 ± 26.5	577.6 ± 44.6		185.1 ± 32.6	161.6 ± 29.2	139.2 ± 25.3	186.5 ± 57.3	
Survival	Sham	461.6 ± 51.6	415.6 ± 45.3	384.7 ± 55.8	420.7 ± 32.8	Stim *p* = 0.59 Region *p* = 0.20 Hem *p* = 0.21 Region × Stim *p* = 0.59 Hem × Stim *p* = 0.16 Region × Hem *p* = 0.39 Region × Hem × Stim *p* = 0.53	55.5 ± 7.0	58.1 ± 13.2	47.3 ± 11.7	42.0 ± 12.3	Stim *p* = 0.22 Region *p* = 0.19 Hem *p* = 0.14 Region × Stim *p* = 0.76 Hem × Stim *p* = 0.08 Region × Hem *p* = 0.13 Region × Hem × Stim *p* = 0.34
	tDCS	444.3 ± 25.0	530.9 ± 49.7	350.3 ± 82.4	450.0 ± 69.5		56.8 ± 12.7	90.4 ± 21.9	66.0 ± 12.1	66.0 ± 10.5	

## Data Availability

Not applicable.

## Supplementary Materials

The following supporting information can be downloaded at https://www.mdpi.com/article/10.3390/ijms24108750/s1.
