# Supplementary material for: Repeated Anodal Transcranial Direct Current Stimulation (RA-tDCS) over the Left Frontal Lobe Increases Bilateral Hippocampal Cell Proliferation in Young Adult but Not Middle-Aged Female Mice
Source: Int J Mol Sci. 2023 May 14;24(10):8750. doi: 10.3390/ijms24108750 (PMC10218697; doi:10.3390/ijms24108750)
Supplement: Supplementary file 1 [file ijms-24-08750-s001.zip › ijms-2365934-supplementary.pdf]

Supplementary Materials: The following supporting information can be downloaded at https://www.mdpi.com/article/10.3390/ijms24108750/s1.

Table S1: Chi-square test values (X^2) as well as significance (p) values obtained regarding the differentiation (in percentages) of newly formed cells that survived in the different regions studied (dHi, vHi) for young adult (2-month-old) and middle-aged (10-month-old) mice. BrdU$^+$/NeuN$^+$/GFAP$^-$, neurons; BrdU$^+$/NeuN$^-$/GFAP$^+$, astrocytes; BrdU$^+$/NeuN$^-$/GFAP$^-$, undifferentiated cells.

	**BrdU$^+$/NeuN$^+$/GFAP$^-$**				**BrdU$^+$/ NeuN$^-$/GFAP$^+$**				**BrdU$^+$/NeuN$^-$/GFAP$^-$**			
	**2 months**		**10 months**		**2 months**		**10 months**		**2 months**		**10 months**	
	**dHi**	**vHi**	**dHi**	**vHi**	**dHi**	**vHi**	**dHi**	**vHi**	**dHi**	**vHi**	**dHi**	**vHi**
**X^2**	0.13	0.49	1.61	0.08	0.25	0.88	6.17	0.11	0.06	0.26	0.04	0.38
**p**	0.72	0.83	0.20	0.77	0.62	0.35	**0.01**	0.75	0.80	0.61	0.84	0.54
